# Could Postnatal Women’s Groups Be Used to Improve Outcomes for Mothers and Children in High-Income Countries? A Systematic Review

**DOI:** 10.1007/s10995-018-2606-y

**Published:** 2018-08-28

**Authors:** Catherine Sikorski, Sietske Van Hees, Monica Lakhanpaul, Lorna Benton, Jennifer Martin, Anthony Costello, Michelle Heys

**Affiliations:** 1 St George’s Hospital, Tooting, London, UK; 2 Institute for Global Health, University College, London, UK; 3 Population, Policy and Practice, UCL Great Ormond Street Institute of Child Health, 30 Guilford Street, London, WC1N 1EH UK; 4 Department of Maternal, Newborn, Child and Adolescent Health, World Health Organization, Geneva, Switzerland

**Keywords:** Postnatal support, Groups, Women’s groups, Participatory approaches, Maternal health, Neonatal health

## Abstract

*Introduction* Participatory postnatal women’s groups have been shown to have a significant impact on maternal and neonatal mortality in low-income countries. However, it is not clear whether this approach can be translated to high-income countries (HICs). We conducted a systematic review to answer the question: “Can postnatal women’s groups improve health outcomes for mothers and children in high-income countries?” *Methods* MEDLINE, EMBASE and Cochrane databases were searched for randomised controlled trials testing any group-based intervention during the postnatal period, in HICs. No limitations were applied to stated outcomes. *Results* Nine trials, including 3029 women, fulfilled the criteria. Group-based interventions, facilitated by health professionals, ranged from didactic to participant-led. Three trials addressed postnatal depression, one addressed physical activity, whilst the remainder looked at multiple health or social outcomes. Three trials reported a significant association between their intervention and at least one outcome measure. Study limitations included poor and inequitable intervention uptake, low participant retention, small sample size and incomplete intervention description. *Discussion* This review found limited and incompletely described evidence testing the use of postnatal group-based interventions to improve health outcomes in HICs. Promising results were reported when the obstacles of sample size and group attendance were overcome. Studies reporting positive impacts on primary outcomes reported higher attendance rates and involved a psychoeducational or cognitive behavioural component in their group approaches. Further research should design and evaluate implementation strategies, assess the use of lay support workers in community settings to improve attendance and retention, and examine the effect of the group environment on outcomes.

## Significance

*What is already known on this subject?* The postnatal period presents a potential opportunity to improve outcomes for mothers and children. Postnatal women’s groups practicing a participatory learning action cycle have been shown in rural low-income settings to improve maternal and neonatal survival.

*What this study adds?* This literature review highlights a lack of well-described, quality studies investigating group-based support for promoting the health and wellbeing of postnatal mothers in high-income countries and the importance of ensuring the logistical challenges of implementing group-based approaches are addressed. The majority of studies did not show an association between the use of post-natal groups and health outcomes. Promising results were found when the obstacles of ensuring attendance are overcome, and where groups involved a psychological component. Currently, insufficient evidence exists to advocate the use of group-based support in the postnatal period in HIC.

## Introduction

The wide-reaching impacts of a woman’s health and health behaviours during the perinatal period present an opportunity for improving health outcomes for both mothers themselves and their children. Maternal health behaviours and health indicators such as smoking (Castles et al. 1999; Knopik et al. 2012; Lindley et al. 2000), alcohol (Andersen et al. 2012; Kesmodel et al. 2002; Sayal et al. 2014) and substance misuse during pregnancy (Cernerud et al. 1996; Chasnoff et al. 1985; Singer et al. 2004; Smith et al. 2008), obesity (Leddy et al. 2008; Ruager-Martin et al. 2010), postnatal depression (Murray et al. 1996; Rahman et al. 2004), and short duration (i.e., less than 3 months) of breastfeeding (Gillman et al. 2001; Howie et al. 1990) have all been associated with a range of poor infant and child health outcomes. In addition, the impact of maternal mental health is not limited to the mother–infant dyad but affects the health and wellbeing of the entire family (Cummings and Davies 1994). Social determinants are strong drivers of maternal health (Collins et al. 1993; Feldman et al. 2000), with socio-economic disadvantage adversely affecting health outcomes (Kahn et al. 2000). Furthermore, Bryant and colleagues describe clear ethnic disparities in maternal health (Bryant et al. 2010). Thus maternal health has physical and social determinants that influence not only the mothers’ own health but also that of their infants, often over the course of their lifetime. The postnatal period, therefore, presents a window of opportunity to improve the health and wellbeing of mothers, their children and potentially the wider family.

In high-income countries (HICs), antenatal support has traditionally been delivered in group form (such as the antenatal classes in the UK provided by the National Health Service and non-governmental organisations such as the National Childbirth Trust). In addition to preparing for childbirth, these meetings set the stage for an informal continuation of peer-to-peer contact beyond the antenatal period, which could be seen as an anecdotal success of the group approach. In contrast, support in the postnatal period, aiming to improve health, nutritional, developmental, and social outcomes, is usually given to mothers on a one-to-one basis, either at home, in a healthcare setting or through telephone calls with a health professional. These postnatal health promotion interventions have shown varying results in the academic literature (Bryanton et al. 2013; Shaw et al. 2006; Fowles et al. 2012; Fu et al. 2014). Interestingly, despite their well-established use during pregnancy, little is known about the potential for group interventions to improve health outcomes during the postnatal period in HIC.

In comparison, in low-income countries (LICs), group interventions adopting a participatory approach, delivered across both the antenatal and the postnatal periods, have improved outcomes for both mothers and babies—even reducing maternal and neonatal mortality (Prost et al. 2013). In their cluster randomised controlled trial of participatory women’s groups in rural areas in eastern India, Tripathy and colleagues found a 31% reduction in neonatal mortality in intervention clusters (Prost et al. 2013; Tripathy et al. 2016). Other trials of similar participatory groups have shown comparable effect sizes (Manandhar et al. 2004). The underlying mechanisms are complex and not completely understood, but are thought to include social support, behaviour change, and, crucially, women’s empowerment (Rath et al. 2010; Younes et al. 2015; Morrison et al. 2010). In LIC, interventions have been largely motivated by reducing neonatal deaths; promoting, for example, the use of clean water, skin-to-skin contact, and keeping infants warm shortly after birth (Kumar et al. 2008).

In the context of financial cuts to public services in some HIC such as the UK, healthcare systems are increasingly recognising the need to learn from low-cost, effective interventions developed in LIC. Group-based support may be more feasible and affordable for local healthcare providers than individual support as it reaches more women and children in each episode. We hypothesised that postnatal support, in the context of a group of women (with or without their partners), could provide differential and perhaps additional benefits compared with individual support. These benefits might arise from sharing challenges with peers to reduce stress and build confidence, learning from coping strategies and best practice within the group to change behaviour and improve the relationship with the healthcare system, and informal support from ongoing local links and friendships beyond the immediate postnatal period. In order to explore this hypothesis further, we carried out a systematic review of postnatal group-based support delivered in HIC. Findings will inform the development of a ‘reverse innovation’ intervention, building on the success of women’s groups in low-income settings. We aimed to gather evidence on different methods and models used, their success, challenges encountered and lessons learned. We asked: “Can postnatal women’s groups (employing participatory and non-participatory approaches) improve outcomes for mothers and children in HIC?”.

## Methodology

### Search Question

For this literature review, we defined the search question using PICOS (Richardson et al. 1995). PICOS is an established model for systematic reviews that breaks the question down into five key elements: Population, Intervention, Comparison, Outcomes, and Study design. Three databases were searched: MEDLINE, EMBASE and Cochrane. Studies were considered to be eligible if they met the following PICOS criteria:

*Population*: HICs.
*Intervention*: any type of group-based support (i.e., both participatory and non-participatory) for women in the postnatal period.
*Comparator*: any.
*Outcomes*: any health or social outcome.
*Study design*: randomised controlled trial.

### Definitions

HIC were defined using the World Bank classification, i.e., any economy with a Gross National Income per capita of $12,476 or more was included in this study (Heys et al. 2016). Group support was framed as any context in which group-based interventions involving postnatal women were being studied. The postnatal period was constrained to the first year after the birth of a child. In studies investigating the impact of women’s groups in low-income settings often draw a distinction between participatory learning and action (a four-phase cycle encouraging participants to: identify and prioritise problems, plan how to address these problems through locally feasible strategies, implement the chosen strategies, and evaluate their activities) and non-participatory interventions (e.g., attending a lecture on healthy birth practices). In this literature review on HIC, both intervention types have been included. The comparators and outcomes were not limited. Lastly, only studies employing a randomised controlled trial design have been included.

### Search Strategy

This literature review was built on a combination of two search terms, “postnatal care” (both the “postnatal” and “post natal” variations) and “group,” and a database-specific filter for randomised controlled trials. For both MEDLINE and EMBASE we used the RCT-filter as developed or adapted by Sign (35). The search was restricted to English language papers with no limitation as to the date of publication. The papers were reviewed independently by three researchers (CS/SvH/MH) and included if they adhered to the PICOS criteria as described above.

**Fig. 1:**
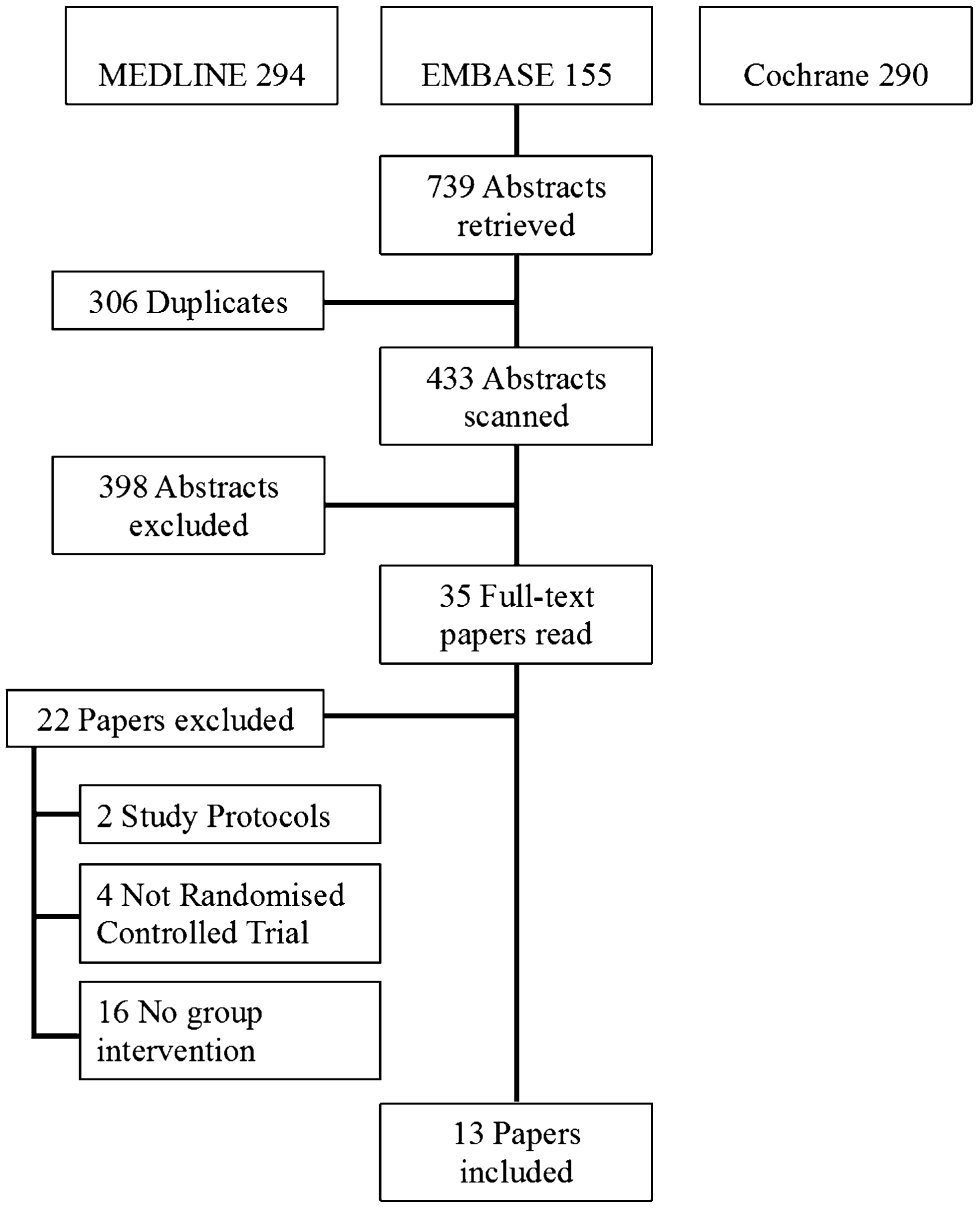
Search strategy and study selection process

The selection process, including the grounds for exclusion, is shown in Fig. 1. In total, 739 citations were retrieved from the MEDLINE, EMBASE and Cochrane databases. After discarding duplicates, 433 abstracts were screened, of which 398 did not meet the inclusion criteria and were excluded. Next, 35 full-text papers were read, of which again 22 did not conform to the criteria. As a result, 13 papers were eligible for inclusion in the final review.

Data from included papers were extracted using a modified CASP (Critical Appraisal Skills Programme, Oxford) (36) worksheet, guided by the PRISMA checklist (Moher et al. 2009), and included article type, funding, location, aim/objective, study design, study date, population (including inclusion/exclusion criteria), recruitment, randomisation, unit of allocation, sample size for each group, withdrawals/exclusions/loss to follow-up, participant characteristics, details of the intervention(s) and any co-interventions, setting in which the intervention was delivered, comparator(s), unit of analysis, form of analysis (intention to treat/per protocol), outcomes (including definitions and measurement tools), statistics used, length and frequency of follow-up, key results, challenges and learning points. The initial searches, study selection and data extraction were performed by a single reviewer (CS) in August and September 2013. Repeat searches, study selection and data extraction were repeated by two reviewers (SvH and MH) between April 2016 and June 2016.

## Results

Although a moderate body of literature exists testing individual forms of postnatal support, such as telephone support (Fu et al. 2014), peer support and home visits (Agrasada et al. 2005), we found only 9 trials, as described in 13 papers, testing group-based postnatal support (Cramp and Brawley 2006, 2009; Escobar et al. 2001; Hagan et al. 2004; Reid et al. 2002; Stamp et al. 1995; Wiggins et al. 2004, 2005; Tandon et al. 2011, 2014; Mendelson et al. 2013; Rouhe et al. 2015; Ryding et al. 2004). These trials utilise diverse frameworks for intervention and investigate varied outcomes among a total of 3029 women. Trials took place in the UK (Reid et al. 2002; Wiggins et al. 2004, 2005), Australia (Hagan et al. 2004; Stamp et al. 1995), Canada (Cramp and Brawley 2006, 2009), Finland (Rouhe et al. 2015), Sweden (Ryding et al. 2004), and the US (Escobar et al. 2001; Tandon et al. 2011, 2014; Mendelson et al. 2013). They are summarised in Table 1.

**Table 1:** Summary of included trials

Study	Location	Primary focus	Intervention	Control	Sample size	Inclusion criteria	Exclusion criteria	Key results	Limitations
Cramp and Brawley (2006, 2009)	Canada	Physical activity (PA)	Group-mediated cognitive behavioural intervention (six sessions immediately following standard postnatal exercise class) at a community-based fitness facility. Focus on developing self-regulatory skills for self-management of physical activity and overcoming postnatal barriers to physical activity	Standard postnatal exercise training programme	57	Women with a (1) primarily sedentary lifestyle, (2) up to a year after giving birth	(1) Long-term medical conditions, (2) currently pregnant, (3) non-English speaking	(1) Group participants reported significantly higher change in frequency, minutes, and volume of PA, (2) group participants’ expectations of the likelihood of achieving their proximal outcomes remained stable, while expectations in the control arm decreased, (3) mean barrier efficacy increased and self-regulatory efficacy remained stable for group participants, while both declined in the control group	(1) Small sample size, (2) participants were self-selected so perhaps unrepresentative of the general postnatal population, (3) participation in PA was self-reported, (4) no follow-up was conducted beyond the length of the intervention itself
Escobar et al. (2001)	US	Health service use and quality of care	Home visits by a nurse (within 48 h after hospital discharge) NB: for the purposes of this review we are interested in the control arm, which includes a group-based intervention	Hospital-based group postnatal education within the first 72 h. The session for up to eight mother–infant pairs was led by a nurse and included physical assessment of the baby, advice on breastfeeding and basic infant care and preventative education	1014	(1) Low medical and social risk (as defined by pre-determined criteria), (2) hospital length of stay was expected to be ≤ 48 h	N/A	(1) No significant differences occurred with respect to: maternal urgent visits, neonatal urgent visits, maternal or neonatal rehospitalisation, breastfeeding discontinuation, or the occurrence of maternal depressive symptoms, (2) mothers in the home visit group were more likely than those mothers in the hospital-based care group to rate multiple aspects of their care as excellent or very good	(1) Studied a socioeconomically low-risk population, (2) only one group session offered, (3) limited power to establish differences between study arms for individual outcomes, (4) poor response rates to questionnaire about maternal satisfaction
Hagan et al. (2004)	Australia	Postnatal depression	A 6-session cognitive behavioural group therapy intervention programme delivered by a research midwife between weeks 2 and 6 after birth	Standard care^a^	199	English-speaking mothers of preterm babies (< 33 weeks) admitted to a neonatal unit	Among others (1) age < 17 years, (2) diagnosis of psychotic disorders, and/or depression, and/or significant substance abuse	No differences between study arms in new diagnoses of depression (using DSM-IV criteria) in the 12 months after delivery	(1) Only half of eligible women participated in the trial, (2) intervention sessions were held when most mothers were still caring for their infant in the neonatal unit
Reid et al. (2002)	Scotland	Physical and mental health, and health service use	Multi-factorial design with two interventions: (1) “Pack”: a postnatal self-help manual, and (2) “Group”: midwife-facilitated weekly support groups, held at community centres. The session agenda was decided with attendees	Not detailed	1004	Among others (1) primiparous women, (2) attending one of two Scottish maternity hospitals	(1) Death of the infant, or (2) admission to SCBU^b^ for > 2 weeks	No significant differences between study arms in scores for three standardised screening tools for postnatal depression, general health and social support. Also no significant differences in health service use	(1) Poor intervention uptake, (2) group attendees were more likely to be middle class and own their home
Rouhe et al. (2015)	Finland	Life satisfaction, wellbeing and healthcare costs for women with fear of childbirth	Group psycho-education led by a psychologist: six sessions during pregnancy and one after delivery	Women received a letter advising them to discuss their fear of childbirth with their usual care provider	371	(1) Nulliparous women, (2) severe fear of childbirth, (3) during first trimester of pregnancy	Not detailed	(1) The groups did not differ in total direct costs, nor in life satisfaction or general wellbeing, (2) SVD^c^ with no complications was registered more often in the intervention group, (3) numbers of non-complicated and complicated caesarean section were higher in the control group	(1) The return rate of self-report questionnaires was low, (2) only one of the sessions took place in the postnatal period, (3) data were not detailed enough to enable cost analysis of antenatal admissions, screening or induction of labour
Ryding et al. (2004)	Sweden	Women after emergency caesarean	Group counselling: two sessions at two months postpartum, with four to five participants. Facilitated by a psychologist and a midwife	Standard care	162	Women giving birth to a live infant by emergency caesarean section at Helsingborg Hospital in Sweden	Non-Swedish speaking women	No difference between groups was found at 6 months postpartum for (1) frightening memories of childbirth, (2) symptoms of posttraumatic stress, (3) postnatal depression	(1) Small sample size, (2) only two intervention sessions offered
Stamp et al. (1995)	Adelaide, South Australia	Postnatal depression	Two antenatal groups (at 32 and 36 weeks gestation) and a postnatal group (6 weeks postpartum) by led a midwife educator. Partners were welcome to attend^d^	Not detailed	144	Women with (1) a singleton pregnancy < 24 weeks’ gestation, (2) vulnerable to postnatal depression, (3) living within the metropolitan area	(1) Non-English speaking women, (2) privately insured women not attending the clinic	No significant difference between the groups’ postnatal depression scores at 6 and 12 weeks and 6 months postpartum	(1) Small sample size, with limited power to detect small changes in depression score, (2) only one of the sessions took place in the postnatal period, (3) poor attendance at groups, particularly in the postnatal period
Tandon et al. (2011, 2014) and Mendelson et al. (2013)	US	Postnatal depression and mood regulation	Standard home visiting services plus the Mothers and Babies (MB) Course: a cognitive behavioural intervention, consisting of six group sessions led by a clinical social worker or clinical psychologist	Standard home visiting services plus information on perinatal depression	78	Women who were (1) pregnant or who had a child less than 6 months of age, (2) enrolled in one of four home visiting programs, (3) at risk for perinatal depression	Women with a current depressive episode	(1) Depressive symptoms declined at a greater rate for the intervention group at 1 week, 3 months and 6 months. However no significant difference was reported between depressive episodes at 6 months, (2) the intervention group experienced greater growth in mood regulation from baseline to 6-month follow-up	(1) Small sample size, with limited power to detect small effects, (2) 28% of women were pregnant at baseline (i.e., at least some of intervention sessions took place antenatally), (3) structured clinical interview to diagnose depressive episode was only conducted at the final 6-month follow-up
Wiggins et al. (2004, 2005)	UK	Several maternal and child health outcomes	(1) Support health visitor (SHV) intervention: seven home visits and additional telephone contacts, (2) community group support (CGS) intervention: community groups for mothers with children < 5 years offering: drop-in sessions, home visiting, and/or telephone support	Standard health visitor services	731	Women living in the Inner London Boroughs of Camden and Islington	Women whose babies (1) had died, (2) were seriously ill, (3) had been placed in foster care	(1) At 12 and 18 months, no significant differences were found between the interventions with respect to: child health, child injury requiring medical attention, infant feeding, maternal smoking levels, and prevalence of maternal depression, (2) SHV women had different patterns of health service use (with fewer taking their children to the GP), and (3) had less anxious experience of motherhood than control women	(1) Poor uptake of services in CGS arm, (2) power to detect effects was limited by presence of two study arms, (3) women who declined to participate were more likely to be from an ethnic minority, (4) uptake of both interventions was lower for women whose first language was not English, (5) the standardised tools used had not been validated in the cultural groups involved in the study

^a^This study included regular biweekly education group sessions

^b^Special care baby unit

^c^Spontaneous vaginal delivery

^d^These groups were offered in addition to the hospital’s antenatal classes

### Study and Participant Characteristics

The studies restricted their sample population using a range of exclusion criteria such as the mothers’ ability to speak English, mothers’ age and infant birth weight. The majority identified and worked exclusively with vulnerable or high risk groups, defined by inner-city disadvantage (Wiggins et al. 2004, 2005; Tandon et al. 2011, 2014; Mendelson et al. 2013), pre-term birth or very low birth weight (Hagan et al. 2004), severe fear of childbirth (Rouhe et al. 2015), having undergone an emergency caesarean section (Ryding et al. 2004), or depression risk (Stamp et al. 1995; Tandon et al. 2011, 2014; Mendelson et al. 2013). The remaining studies, i.e., three out of a total of nine trials, looked at mother–infant pairs at low risk of developing postpartum complications (Cramp and Brawley 2006, 2009; Escobar et al. 2001; Reid et al. 2002), of which one study actively excluded high-risk groups (Escobar et al. 2001).

### Participant Recruitment to Studies

Recruitment was carried out in a number of locations, such as at antenatal clinics (Reid et al. 2002; Stamp et al. 1995), at local hospitals (Rouhe et al. 2015; Ryding et al. 2004), on postnatal wards of hospitals (Escobar et al. 2001; Hagan et al. 2004), through home-visiting programmes (Tandon et al. 2011, 2014; Mendelson et al. 2013), and via local newspapers (Cramp and Brawley 2006, 2009) and birth records (Wiggins et al. 2004, 2005).

#### Intervention Logistics

##### Postnatal Versus Antenatal Delivery of Groups

All but three groups started meeting after birth (Stamp et al. 1995; Tandon et al. 2011, 2014; Mendelson et al. 2013; Rouhe et al. 2015). However, of these aforementioned three studies, the majority of the groups still took place during the postnatal period. The one exception was Rouhe and colleagues’ trial comparing measures of wellbeing among nulliparous women with fear of childbirth, in which only one of the six psychoeducational group sessions took place after delivery (Rouhe et al. 2015). One study looking to reduce depressive symptoms did not distinguish between the ante- and postnatal periods; they simply included women who were either pregnant or who had a child less than 6 months of age (Tandon et al. 2011, 2014; Mendelson et al. 2013).

##### Group Location

The location for the group interventions were not always specified, but where described women met in community centres (Reid et al. 2002), hospitals (Escobar et al. 2001; Hagan et al. 2004) and a gym (Cramp and Brawley 2006, 2009).

##### Number and Duration of Group Sessions

Details of the numbers and duration of groups were provided in six of the nine trials. A similar number of group sessions (between six and seven) took place across most studies, although one offered only a single group meeting (Escobar et al. 2001). In contrast, the reported duration of group sessions varied widely (from 20 min to 2 h). Thus total potential exposure time to a group intervention, where reported, varied from 120 min (Cramp and Brawley 2006, 2009; Escobar et al. 2001) to a maximum of 420 min (Hagan et al. 2004; Reid et al. 2002; Tandon et al. 2011, 2014; Mendelson et al. 2013; Rouhe et al. 2015; Ryding et al. 2004) assuming optimal attendance.

##### Intervention Strategies

Intervention formats included one-off meetings (Escobar et al. 2001), drop-in sessions (Wiggins et al. 2004, 2005) and structured groups meeting twice a week (Cramp and Brawley 2006, 2009), weekly (Hagan et al. 2004; Reid et al. 2002; Tandon et al. 2011, 2014; Mendelson et al. 2013) or at fortnightly (or longer) intervals (Stamp et al. 1995; Rouhe et al. 2015; Ryding et al. 2004). Facilitators, where specified, were trained professionals and came from midwifery (Hagan et al. 2004; Reid et al. 2002; Stamp et al. 1995; Ryding et al. 2004), nursing (Escobar et al. 2001), psychology (Tandon et al. 2011, 2014; Mendelson et al. 2013; Rouhe et al. 2015; Ryding et al. 2004), social work (Tandon et al. 2011, 2014; Mendelson et al. 2013) and community care (Wiggins et al. 2004, 2005) backgrounds. Two studies did not describe the background of their group facilitators (Cramp and Brawley 2006, 2009). Topic frameworks focused on the baby (Escobar et al. 2001), or on the mother (Cramp and Brawley 2006, 2009; Hagan et al. 2004; Stamp et al. 1995) or emphasised the mother–baby relationship (Mendelson et al. 2013). Delivery styles varied from didactic (Cramp and Brawley 2006, 2009; Escobar et al. 2001) to participant-led (Reid et al. 2002; Stamp et al. 1995; Ryding et al. 2004) or combined didactic instruction with group activities and discussion (Hagan et al. 2004; Tandon et al. 2011, 2014; Mendelson et al. 2013). Three trials made use of group-based cognitive behavioural therapy (Cramp and Brawley 2006, 2009; Hagan et al. 2004; Tandon et al. 2011, 2014; Mendelson et al. 2013), one of which (Tandon et al. 2011, 2014; Mendelson et al. 2013) employed the Mothers and Babies (MB) Course, a cognitive behavioural intervention designed to reduce the risk of postnatal depression by promoting maternal self-efficacy and positive mood states (Muñoz et al. 2007). One study engaged both the control and intervention groups in group-based care (Stamp et al. 1995). Routine antenatal classes offered by the hospital served as the control group. Even though these classes did not include specific information about the outcome measure of postnatal depression until the last week of the intervention (i.e., 6 weeks postpartum), Stamp and colleagues’ findings cannot be simply linked to the implementation of group-based support.

##### Intervention Uptake and Group Attendance Rates

Only four papers described the number of participants attending group sessions, which ranged from a minimum or 4 (Ryding et al. 2004) up to 10 participants (Escobar et al. 2001; Stamp et al. 1995). In Scotland, Reid et al. reported that most support groups had fewer than four women in attendance, and 89 of 309 sessions could not be run as no one attended (Reid et al. 2002). Overall, the uptake of the group-based interventions was low, for example Reid and colleagues reported a participation rate as low as 18% for the support groups (Reid et al. 2002) and in Wiggins and colleagues’ study only 19% of the women allocated to the intervention took part in the community groups (Wiggins et al. 2004, 2005). One intervention actively encouraged the participation of partners or other supportive individuals, but this study also reported low attendance (31%) (Stamp et al. 1995).

##### Outcome Measures

Four of the studies focussed on single health outcomes—three of which looked at postnatal depression (Hagan et al. 2004; Stamp et al. 1995; Tandon et al. 2011, 2014) and one at physical activity among postnatal women (Cramp and Brawley 2006, 2009). The latter study on physical activity also assessed a number of additional process measures such as proximal outcome expectations and factors of group cohesion and collaboration (Cramp and Brawley 2006, 2009). The remainder looked at multiple health outcomes or a broader scope of measures including breastfeeding discontinuation (Escobar et al. 2001), postnatal depression (Escobar et al. 2001; Reid et al. 2002; Wiggins et al. 2004, 2005; Ryding et al. 2004), the level of fear after childbirth (Ryding et al. 2004), mood-regulation (Mendelson et al. 2013), life satisfaction and general wellbeing (Rouhe et al. 2015), smoking (Wiggins et al. 2004, 2005), social support (Reid et al. 2002; Mendelson et al. 2013), health service use (Escobar et al. 2001; Reid et al. 2002) and costs.

##### Findings

Of the nine trials, three reported a significant effect of the intervention on at least one primary outcome measure (Cramp and Brawley 2006, 2009; Tandon et al. 2011, 2014; Mendelson et al. 2013; Rouhe et al. 2015). All of these ‘significant’ trials employed a psychoeducational or cognitive behavioural component to their intervention. Tandon et al. (2011, 2014) reported a greater rate of reduction of depressive symptoms among low-income women randomised to attend the MB Course. Reporting on the same trial, Mendelson et al. (2013) showed a greater growth in mood regulation (i.e., the belief that one is able to adjust negative mood to a more positive emotional state) at 6-months’ follow-up among women in the MB Course trial arm (Mendelson et al. 2013). Cramp and Brawley (2006, 2009) found a significant effect of their group-based cognitive behavioural intervention on exercise behaviour change, and an increase in the frequency and volume of physical activity. Lastly, Rouhe et al. (2015) found that nulliparous women with a fear of childbirth who attended their psychoeducational group were more likely to have a spontaneous vaginal delivery with no complications. Also, fewer of these women with group sessions had additional visits to specialist maternity care. However, in this study only one of the group sessions, of the maximum seven on offer, was held during the postnatal period. The other six trials reported no significant differences between trial arms (group-based vs. no group-based support) in primary outcome measures. Possible reasons for the lack of statistical significance, which we explore further in our discussion, include poor intervention uptake (Cramp and Brawley 2009; Hagan et al. 2004) and small sample sizes (Escobar et al. 2001; Reid et al. 2002; Stamp et al. 1995; Wiggins et al. 2005; Rouhe et al. 2015).

## Discussion

A clear gap exists in the literature with respect to group-based interventions for postnatal women in HICs. In the UK, the need for high quality evidence for postnatal support services has recently been highlighted by the opportunity presented by the Perinatal Mental Health Community Services Development Fund in the UK, which aims to improve access to community mental health services for pregnant women and new mothers experiencing mental health difficulties.

In our review, only 13 articles, describing 9 trials, met the PICOS criteria of being set in HIC, involving group-based support for postnatal women, and employing a randomised controlled trial study design. In addition, the research that has been done to date reveals a number of often-encountered difficulties in the organisation and implementation of group-based experiments. The trials of group-based interventions that reported a positive impact on their primary outcomes commonly involved a psychoeducational or cognitive behavioural component and reported higher attendance rates.

### Limitations to the Studies

A number of methodological concerns were identified in the reviewed studies. First, poor or inequitable uptake of and attendance at the groups was a recurring concern, with some researchers attributing their lack of significant results directly to low attendance rates (Reid et al. 2002; Stamp et al. 1995; Wiggins et al. 2004, 2005). Reid and colleagues demonstrated that the postnatal women who attended one group meeting were likely to return (Reid et al. 2002). Satisfaction, however, may not equate with effectiveness. Hagan et al. reported no association between the number of sessions attended and the development of postnatal depression (Hagan et al. 2004). High attendance rates were seen where groups were predominantly delivered in the antenatal period—for example in Rouhe et al. (2015). Interestingly, in this latter study, no apparent attendance-boosting strategies were being used—however the study itself tackled the issue of fear of childbirth. Excellent attendance and study retention rates were also described by Tandon and colleagues who provided transport, childcare (if needed), a reminder email or phone call and a meal at each session for participants (Tandon et al. 2011, 2014). Their trial reported statistically significant reductions in depressive symptoms.

Second, perhaps as a result of poor retention rates, underpowered sample sizes were a barrier to reaching statistical significance (Escobar et al. 2001; Stamp et al. 1995; Mendelson et al. 2013; Ryding et al. 2004). In one study, among the 506 mother–infant pairs randomised to the group-support arm, only 157 received group support alone, 264 attended an individual hospital visit, and 64 had both an individual hospital visit and group support (Escobar et al. 2001).

Third, concerns regarding the largely suboptimal recruitment and retention rates were compounded in at least one trial by inequitable uptake. Reid et al. (2002) explicitly linked socio-economic status to intervention uptake, reporting a higher proportion of women from middle class than working class backgrounds attending postnatal support groups (38 vs. 17%, with ‘class’ defined by occupation and postcode deprivation score). These concerns limit the generalisability of study findings and interventions. The researchers explained this social class bias by alluding to the better resources and/or greater social confidence of middle class women. This form of bias is an ongoing concern in public health and health interventions where inequitable uptake of services and interventions may then lead to a widening of health inequalities. Interestingly one of the three trials reporting positive outcomes, which also employed strong recruitment and retention incentives, preferentially recruited low-income African American women (83.1%) (Tandon et al. 2011, 2014). The majority of these women were unmarried (approximately 78%) and unemployed (approximately 70%).

Fourth, most studies followed up participants for a relatively short follow-up time (ranging from 2 weeks (Escobar et al. 2001) to 18 months Wiggins et al. 2004, 2005) with only two trials following up participants beyond 6 months postpartum (Hagan et al. 2004; Wiggins et al. 2004, 2005). Thus the question of sustained impact cannot be addressed in most—if not all—of the studies. Cramp and Brawley consider the duration of their study (i.e., 8 weeks) to be one of its limitations (Cramp and Brawley 2006, 2009). They suggest that future research explores whether the significant increase in physical activity of women in the cognitive behavioural intervention-group is sustained for a longer period of time after giving birth.

Finally, it is important to underline that the finding that three of the nine reported trials rendered significant results, does not imply that the group-based element was the (sole) critical factor in yielding a significant impact on the participating women. These being complex interventions, it remains unclear which of the many variables, for example outcome of choice, group size, participants, session content or methodology, timing or setting of the meetings were the key impetus behind the significant results. As such, simply to hail a group-based therapy as successful or otherwise without a thorough evaluation of the implementation strategy and wider context would ignore the many other variables at play in these interventions. Furthermore, the interventions had different objectives, and although this literature review intentionally did not distinguish between outcome measures, we need to be mindful that we are comparing studies that were set up with disparate aims.

### Limitations to Systematic Review

The distinctions between these nine trials make it hard to draw generalising conclusions about the potential for postnatal group-based therapies to prompt positive health outcomes in HIC. The studies were heterogeneous in outcome measures and study population. They considered the effect of postnatal groups on both physical and mental health—arguably some health outcomes will be relatively more straightforward to affect than others. Moreover, the trials adopted a variety of frameworks for intervention, ranging from didactic to methodologically structured, and to participant-led. Finally, these trials investigated a total of 3029 diverse women from a wide range of socioeconomic, ethnic and educational backgrounds.

In this review, we intentionally included only RCTs in order to develop the most robust evidence base. Of course other methods of evaluation may also provide useful insights into efficacy and intervention development, and by excluding these studies we may have lost some information. However only four relevant studies found in our database searches were excluded due to study design. We intentionally did not restrict our review based on outcome measures. For three of the studies (Reid et al. 2002; Wiggins et al. 2004; Tandon et al. 2011, 2014; Mendelson et al. 2013) the intervention took place in both the antenatal and postnatal periods, limiting the extent to which we can attribute any effect to the postnatal component of the intervention for the purposes of our review.

### Focus for Future Research

There have been some promising results that are worthy of further research. First, the MB Course aiming to reduce the risk of postnatal depression rendered significant findings (Tandon et al. 2011, 2014; Mendelson et al. 2013). The MB programme consists of six 2-h cognitive behavioural therapy sessions delivered weekly in a group. In similar fashion, Cramp and Brawley integrated a cognitive behavioural intervention into their postnatal exercise programme yielding significant results (Cramp and Brawley 2006, 2009). Of note, each of these group-based interventions met at least once a week over a period of 4–6 weeks. Second, all three trials with significant results had a predefined methodology, with Rouhe and colleagues being the exceptions in investigating not a cognitive behavioural but a psychoeducational group method (Rouhe et al. 2015). Arranging for transport, childcare, and a meal at each session seemed to reduce the loss to follow-up of low-income participants in comparison to studies where the women had to arrange their own means (Tandon et al. 2011, 2014). Importantly, none of the research groups appeared to employ a framework that involved co-production. The questions of whether participant involvement in the intervention design might lead to greater success and/or that postnatal group approaches could be tailored and successful in women only with high psychosocial risk is one that should be addressed further.

## Conclusion

This systematic literature review highlights the lack of studies investigating group-based support for promoting the health and wellbeing of postnatal mothers in HICs. Significant operational challenges are likely to have contributed to the lack of data. These challenges involve logistical barriers inherent to group work, particularly relating to attendance, and small effect sizes due to underpowered sample sizes. Currently, insufficient evidence exists therefore to advocate the use of group-based support in the postnatal period in HIC. However, this literature review has found promising results within the postnatal, group RCT literature when the obstacles of securing statistically effective sample sizes and ensuring attendance are overcome. Despite these findings and in the context of financial pressures currently sustained in health services across Europe, there remains a need to learn from successes in resource-poor settings. Further research is needed to assess the core components of models of care found to be effective in LIC that could be adapted and applied to HIC.
